# Personality, Emotion Regulation, and Psychological Distress in Italian Women with Feeding and Eating Disorders: A Cross-Sectional Study

**DOI:** 10.3390/healthcare14111517

**Published:** 2026-05-29

**Authors:** Maria Rosaria Juli, Laura Muzi, Valentina Tavoloni, Mariagrazia Di Giuseppe

**Affiliations:** 1 Department of Health Sciences, Magna Graecia University of Catanzaro, 88100 Catanzaro, Italy; mariarosaria.juli@unicz.it; 2 Department of Philosophy, Social Sciences, Humanities and Education, University of Perugia, 06123 Perugia, Italy; laura.muzi@unipg.it; 3 Department of History, Humanities and Society, University of Rome Tor Vergata, 00133 Rome, Italy; tavoloni.valentina@gmail.com

**Keywords:** feeding and eating disorders, purging, psychological distress, emotion regulation, personality

## Abstract

**Highlights:**

**What are the main findings?**

- Negative affectivity, detachment, and purging symptoms emerged as significantly associated with psychological distress in patients with FEDs.
- Emotion regulation showed an indirect effect on the relationship between self-esteem and purging symptoms in patients with FEDs and Obesity.

**What are the implications of the main findings?**

- Particular attention should be paid to specific aspects of personality functioning when planning tailored treatment with FED patients.
- Therapeutic intervention should consider the impact of both explicit and implicit components of emotional regulation on the symptomatology of FEDs. With particular reference to purging symptoms, understanding the role of emotion regulation could provide important insights about how to intervene for a more effective treatment.

**Abstract:**

**Background/Objectives**: Feeding and Eating Disorders (FEDs) are among the most challenging mental disorders due to their pervasive symptomatology and high relapse rates. While considerable research has focused on the role of personality in the onset and maintenance of FEDs, it remains unclear whether specific personality dimensions and emotion dysregulation mechanisms predict clinical severity and purging behaviors. This study aimed to explore the role of personality dimensions, emotion dysregulation, and purging behaviors in predicting psychological distress in patients with FEDs, adopting a dimensional and integrated perspective. **Methods**: A sample of cisgender women in a semi-residential treatment for FEDs or obesity (*n* = 124) was recruited in southern Italy and assessed using a psychodiagnostics survey, including the Eating Disorder Inventory-3 (EDI-3), the Symptom Checklist-90-R (SCL-90-R), and the Personality Inventory for DSM-5 (PID-5). **Results**: Patients with bulimia nervosa exhibited higher psychological distress compared to patients with other FEDs and Obesity, which was not significantly determined by the co-occurrence of personality disorders. Negative affectivity, detachment, and purging symptoms were significantly related to psychological distress in patients with FEDs and Obesity (*p* ranging from 0.028 to <0.001). Moreover, the results showed an indirect effect of emotion regulation on the relationship between self-esteem and purging symptoms in patients with FEDs and Obesity (*β* = 0.107; *p* = 0.046). **Conclusions**: These findings suggest that specific personality dimensions, emotion dysregulation, and purging symptoms are associated with psychological distress in individuals with FEDs and Obesity. Therefore, it is necessary to reflect on the impact of these psychological components in planning tailored treatment for FED patients.

## 1. Introduction

Feeding and Eating Disorders (FEDs) are complex, multifactorial psychopathological conditions characterized by persistent alterations in eating behavior, excessive and pervasive concern about body weight and shape, and significant impairment in psychological, emotional, and psychosocial functioning [1]. They mainly arise in adolescence and early adulthood, but in recent years, a decreased age of onset has been reported [2]. FEDs mainly affect females, although recent evidence indicates a progressive rise in their prevalence among young males [3,4]. The overall prevalence of FEDs in adolescence ranges from 5.5% to 18% in females and from 0.6% to 2.4% in males, with considerable heterogeneity across diagnostic subtypes. Recently, increasing attention has been directed toward the relationship between FEDs and Obesity [5]. Although the Diagnostic and Statistical Manual 5th edition Text Revised (DSM-5 TR; [6]) does not include Obesity as a FED, a large body of literature documents the high comorbidity and overlap between this pathological condition and dysfunctional eating behaviors, particularly in the context of BED. Epidemiological and clinical studies have shown that BED is frequently present in obese samples and that difficulties in emotional regulation represent a shared psychopathological mechanism between obesity and eating disorders [7]. Consistent with this evidence, some contributions highlight how obesity and eating disorders share aspects of clinical presentation and require integrated assessment approaches [8]. Finally, similar psychopathological profiles are highlighted between subjects with BED and obese individuals in clinical settings [9].

In addition to severe functional impairment, patients with FEDs show high emotional suffering that includes symptoms of anxiety, depression, affective instability, and interpersonal difficulties [10,11]. Overall, psychological distress has been found to be significantly more prevalent and pervasive in patients with FEDs compared to the general population [12]. In particular, research has suggested that FEDs characterized by binge episodes and purging behaviors show higher levels of psychological distress than restrictive subtypes, especially regarding depression, anxiety, and relationship difficulties [13,14,15]. BN is frequently described as the subtype most associated with emotional instability, impulsivity, and impaired psychosocial functioning. Clinical studies [16,17] show that individuals with BN exhibit high levels of impulsive traits such as negative urgency and a lack of planning, which are correlated with symptom severity and the presence of comorbid psychopathology.

In light of this evidence, growing interest has been directed toward the role of personality in understanding the psychopathology of FEDs [18,19]. The contemporary dimensional model of the DSM-5-TR, the Alternative Model for Personality Disorders (AMPD), conceives of personality psychopathology along a continuum of adaptive–maladaptive functioning rather than as discrete diagnostic categories [20,21]. Numerous studies have documented a high prevalence of personality disorders in patients with FEDs [22,23,24]. However, the mere presence of a categorical diagnosis of personality disorder does not appear sufficient to explain the clinical heterogeneity and levels of psychological distress observed in FEDs, suggesting the need to adopt a dimensional approach to personality functioning. Although extensive research has examined the co-occurrence of personality disorders (PDs) and FEDs [22,25], the investigation of disordered eating through the PID-5 framework remains in its early stages. From this perspective, specific personality dimensions—such as negative affectivity, interpersonal detachment, and disinhibition—have been identified as transdiagnostic factors associated with psychological distress, symptom severity, and clinical heterogeneity of FEDs [26,27]. Furthermore, personality traits distinctively shape eating symptomatology. The empirical literature indicates a positive association between restrictive eating and rigidity, perfectionism, and social avoidance [22,28,29], whereas impulsivity and low mood are linked to binge eating behavior. More broadly, specific personality domains (i.e., negative affectivity, disinhibition, psychoticism, antagonism, and detachment) represent a vulnerability factor for psychological distress, which, in turn, may contribute to the exacerbation of eating symptomatology [30,31].

Relatedly, a key role is played by emotional dysregulation, defined as a persistent difficulty with identifying, modulating, and tolerating affective states, as well as adopting adaptive strategies to cope with emotions that are perceived as intense or threatening [32]. In FEDs, emotional dysregulation is closely related to both general psychopathology and the use of dysfunctional eating behaviors. In particular, binge episodes and purging behaviors may serve a maladaptive emotional regulation function, providing temporary relief from emotional distress [14,33,34]. Empirical evidence suggests that intrapsychic variables such as low self-esteem, negative affectivity, and emotional vulnerability contribute indirectly to the emergence of purging behaviors through an increase in emotional dysregulation [35,36,37]. From this perspective, purging can be conceptualized not only as a symptom of specific FEDs but also as a behavioral expression of a broader difficulty in modulating intense affective states, often in interaction with specific dysfunctional personality dimensions [38]. Overall, the literature suggests that understanding FEDs requires an integrated approach that gives consideration beyond eating symptomatology, psychological distress, personality, and emotion regulation processes. In addition to explicit emotion regulation processes, recent literature has highlighted the role of implicit regulatory mechanisms in shaping emotional responses and psychological adaptation in clinical populations. These processes may contribute to the maintenance of maladaptive behaviors and to the overall level of psychological distress [39,40,41]. However, it remains unclear as to whether the presence of personality disorders per se contributes to greater distress in FEDs, or whether specific personality dimensions and emotion dysregulation mechanisms better predict clinical severity and purging behaviors. In light of these gaps, the present study aims to explore the role of personality dimensions, emotion dysregulation, and purging behaviors in predicting psychological distress in patients with FEDs, adopting a dimensional and integrated perspective.

### Hypotheses

This research was organized around four hypotheses aimed at investigating whether (1) patients with BN would exhibit higher psychological distress compared to patients with other FED diagnoses; (2) the majority of patients with FEDs would show comorbidity with personality disorders (PDs), and whether such comorbidity would be associated with higher psychological distress compared to patients with only FEDs; (3) personality dimensions and purging symptoms would relate to psychological distress in patients with FEDs; and (4) emotion regulation would show an indirect effect on the relationship between self-esteem and purging symptoms in patients with FEDs.

## 2. Materials and Methods

### 2.1. Sample

The present study enrolled 124 female patients diagnosed with FEDs receiving semi-residential treatment at a National Health Service center for FEDs in Southern Italy. Patients were recruited from September 2025 to January 2026 and included both new admissions and patients already in treatment. Inclusion criteria were as follows: (a) a FED diagnosis formulated according to DSM-5-TR criteria by the multidisciplinary clinical team; (b) age 11 years and older, consistent with the acceptance rules of the National Health Service center for FEDs in which the sample was recruited; and (c) willingness to participate in the research, expressed via signed informed consent. Patients with (a) severe cognitive impairments or (b) acute psychopathological conditions that prevented them from understanding and completing the questionnaires independently were excluded from the research.

Patients’ mean age was 28.3 years on average (*SD* = 13.3), ranging from 11 to 67 years. The mean duration of illness was 12.7 years (*SD* = 12.3), ranging from 0 to 46 years since the first FED diagnosis. The most prevalent FED according to the DSM-5-TR approach was bulimia nervosa (BN; *N* = 41; 33.1%), followed by anorexia nervosa (AN; *N* = 29; 23.4%), Binge Eating Disorder (BED; *N* = 23; 18.5%), and OSFED (*N* = 7; 5.6%). The remaining 19.4% of the sample was represented by patients with a diagnosis of obesity (OB; *N* = 24). The majority of FED patients (*N* = 74; 59.7%) also received a diagnosis of comorbid PD, which was distributed among FEDs as follows: BN 18.6% (*N* = 23), AN 13.7% (*N* = 17), BED 12.1% (*N* = 15), Obesity 11.3% of (*N* = 14), and OSFED 4.0%.

### 2.2. Measures

The present cross-sectional study employed the Italian validated version of self-reported questionnaires to assess eating disorder symptoms, personality dimensions, emotional dysregulation, self-esteem, and overall psychological distress in patients with FEDs. Despite the length of the survey, none of the participants reported any notable signs of fatigue in completing it.

The *Eating Disorder Inventory-3* (EDI-3) [42] is a 91-item self-report questionnaire assessing the presence and intensity of clinically relevant symptoms in FEDs on a 6-point Likert scale. The EDI-3 represents an improvement of the earlier versions of the scales and includes three subscales measuring eating disorder symptoms, such as drive for thinness (DT), bulimia (B), and body dissatisfaction (BD), and nine relevant psychological trait subscales, such as low self-esteem (LSE), personal alienation (PA), interpersonal insecurity (II), interpersonal alienation (IA), interoceptive deficits (ID), emotional dysregulation (ED), perfectionism (P), asceticism (AS), and maturity fear (MF). It is used in both clinical and research settings for screening, supporting clinical diagnosis, and planning treatment since it shows excellent reliability (Cronbach’s *α*  = 0.90–0.97; test–retest *r*  =  0.98) [42,43]. In the current study, Cronbach’s *α* for the EDI-3 was 0.92.

The *Symptom Checklist-90-R* (SCL-90-R) [44] is a 90-item self-report questionnaire that is widely used to measure a range of psychological and psychiatric symptoms. It assesses nine primary symptom dimensions on a 5-point Likert scale, including somatization (SOM), obsessive-compulsive (O-C), interpersonal sensitivity (IS), depression (DEP), anxiety (ANX), hostility (HOS), phobic anxiety (PANX), paranoid ideation (PI), and psychoticism (PSY). Seven additional items are also included to assess appetite and sleep disturbances. Furthermore, the scale contains three global indices designed to provide indicators of symptom severity and psychological distress. In particular, the Global Severity Index (GSI) indicates the intensity of the psychological distress reported by the subject and is widely used as an outcome measure in clinical research. The SCL-90-R has been shown to have good reliability and high internal consistency for all subscales (α = 0.70). Validity and reliability of the scale are well-documented [45,46]. In the current study, Cronbach’s *α* for the SCL-90-R was 0.77.

The *Personality Inventory for DSM-5* (PID-5) [47] is a 220-item self-report questionnaire assessing pathological personality traits on a 4-point Likert scale according to the AMPD of the DSM-5-TR Section III [6]. The PID-5 assesses 25 facet traits that are organized into five higher-order domains: negative affectivity, detachment, antagonism, disinhibition, and psychoticism. These domains represent transdiagnostic dimensions of personality functioning and are conceptually overlapping, but not identical, to the main pathological personality factors described in the literature. The PID-5 showed adequate internal reliability at both the domain and facet levels (Cronbach’s *α*  > 0.70 for all facet scales and >0.90 for all domain scales [48]), as well as robust convergent and discriminant validity in both clinical and non-clinical samples [47,49]. In particular, the dimensional approach of the PID-5 is particularly useful in complex clinical conditions, such as FEDs, in which dysfunctional personality traits can manifest along a continuum and across diagnostic categories. In the current study, Cronbach’s *α* for the PID-5 was 0.84.

### 2.3. Procedure

After receiving a detailed explanation of the study’s objectives and procedures, all eligible patients were invited to voluntarily participate anonymously. Those who provided written informed consent were administered a battery of self-report questionnaires and completed the survey in a dedicated environment under the supervision of trained clinical staff. For minor participants, who were asked to complete the same survey administered to patients above 18 years of age, informed consent was signed by both parents and legal guardians, in addition to the minor’s informed consent. The average time to complete the questionnaire was approximately 40–50 min. Participation in this study did not entail any changes to the planned diagnostic or therapeutic pathway, and no incentives for participation were provided. Data were collected and processed in compliance with current legislation regarding privacy and personal data protection. Furthermore, this study was conducted in accordance with the ethical principles of the Declaration of Helsinki and was approved by the local Ethical Committee [Ethical Committee of the Region Calabria (Protocol N.189 registered in 13 June 2024)]. The information sheet and the participant informed consent form were also prepared as required by the ICH Guidelines. None of those who agreed to participate subsequently dropped out of this study.

### 2.4. Statistical Analyses

The non-normal distribution of the studied variables has been verified by applying the Shapiro–Wilk Test (W ranging from 0.657 to 0.898; all *p* < 0.001). Age was statistically controlled to avoid confounding effects. To analyze whether FEDs differed from one another in psychological functioning, we performed a Kruskal–Wallis test with DSCF pairwise comparisons on the EDI-3 composite scores. To test whether comorbidity with PDs was associated with significantly higher psychological distress compared to patients with only FEDs, we performed a Mann–Whitney U test. To investigate whether personality dimensions and purging symptoms predicted psychological distress in patients with FEDs, we performed multiple linear regression analyses. To evaluate whether emotion regulation mediated the effect of self-esteem on purging symptoms, we used the General Linear Model (GLM) Mediation Analysis. All analyses were performed with the statistical software Jamovi Version 2.4.

## 3. Results

### 3.1. Differences in Psychological Responses and Adjustment Among Patients with Feeding and Eating Disorders and Obesity

Descriptive statistics showed high scores in all composite subscales of the EDI-3, as reported in our sample, and are displayed in Table 1. Bulimic patients showed the highest scores in ineffectiveness, interpersonal problems, affective problems, overcontrol, and global psychological maladjustment, as compared to patients with other FED or obesity diagnoses. We performed Kruskal–Wallis analysis to test whether FEDs differed from one another in these psychological dimensions. The results showed that interpersonal and affective problems, overcontrol, and global psychological maladjustment were significantly different among clinical subtypes (*p* ranging from 0.019 to 0.006). DSCF pairwise comparisons revealed that only obese patients differed significantly from AN and BN in all analyzed scales, while no differences were found among other FEDs.

### 3.2. Psychological Distress in Patients with Feeding and Eating Disorders with or Without Personality Disorders

PDs were found to be highly prevalent in our sample. More than half of the participants also received a PD diagnosis (*N* = 74; 59.7%), with no significant differences between clinical subtypes. The co-occurrence of PD in patients with FEDs and Obesity is displayed in Table 2. A Mann–Whitney U test was performed to investigate differences in psychological dimensions and symptoms among patients with (group 1) or without (group 2) comorbid PD diagnosis. The results showed no significant differences between the two groups, except for the SCL-90-R Depression subscale (*p* = 0.023), on which FED and Obese patients with comorbid PDs showed greater scores (*X_DEP_* = 103.6; *SD* = 15.2) than FED and Obese patients without co-occurring PD.

### 3.3. Personality Domains and PURGING Symptoms as Predictors of Psychological Distress in Feeding and Eating Disorder Patients

The mean scores and standard deviations obtained for the five personality domains, such as negative affectivity, detachment, antagonism, disinhibition, and psychoticism, are displayed in Table 3. Our sample’s scores were consistent with the average scores found in other clinical samples [49], with only antagonism and disinhibition slightly below the reference values.

Personality facet traits, assessed with the PID-5, and purging symptoms, assessed with the corresponding subscale of the EDI-3, were entered into the linear regression model as independent variables, while the SCL-90-R GSI was included as a dependent variable measure of overall psychological distress. The model was significant and explained 65% of the variance (R^2^ = 0.739; Adjusted R^2^ = 0.657; F = 8.99; *p* < 0.001). However, only negative affectivity, detachment, and purging symptoms emerged as significant predictors of the GSI. Regression coefficients are displayed in Table 4.

**Effect of self-esteem on purging symptoms mediated by emotion regulation**

**Figure 1:**
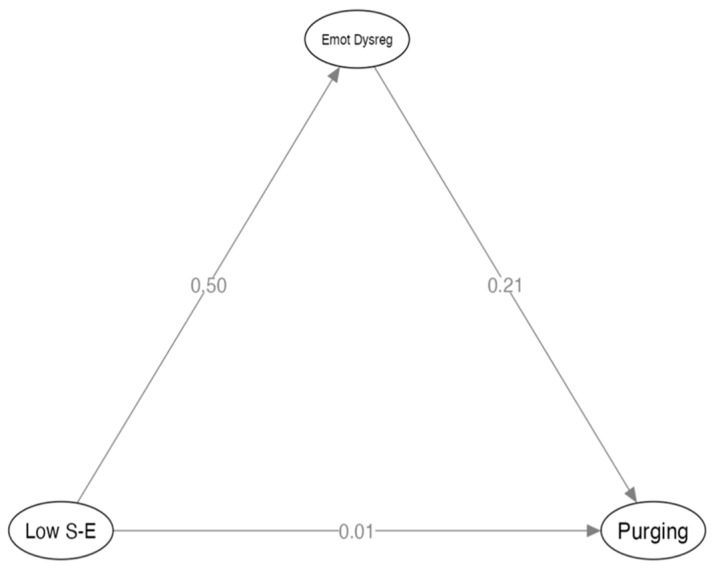
The path model of emotion regulation mediating the effect of self-esteem on purging symptoms. Note: Low S-E = low self-esteem; Emot Dysreg = emotional dysregulation; Effects are displayed as standardized scores (Betas).

The path models of the GLM mediation analyses are displayed in Figure 1. The results are displayed in Table 5 and reveal no direct statistical association of low self-esteem with purging symptoms, assessed with the EDI-3 LSE and B component scales, respectively, which instead contributed to increased emotion dysregulation by 0.50 standard deviations for every standard deviation increase in low self-esteem (*p* < 0.001). The direct statistical association of emotion dysregulation with increasing purging symptoms was also significant (*p* = 0.036). Additionally, emotion dysregulation mediated (indirect; *p* = 0.046) the relationship between low self-esteem and purging symptoms, resulting in an increase of 0.012 standard deviations in purging symptoms for every standard deviation increase in low self-esteem.

## 4. Discussion

The clinical presentations of FEDs involve a highly complex constellation of symptoms that encompasses eating behaviors, personality features, and emotion regulation. The present study investigated the impact of purging behaviors and emotion dysregulation on psychological distress experienced by patients with FEDs and Obesity with or without comorbid personality disorders. This research was mainly inspired by empirical evidence about the role of emotional dysregulation in the onset and course of various mental disorders [50,51,52,53,54,55,56].

The first hypothesis, that BN was associated with higher psychological distress than other FEDs, was partially confirmed. Findings revealed that BN patients reported the highest levels of ineffectiveness, affective and interpersonal problems, overcontrol, and overall psychological maladjustment. While significant differences on these psychological aspects were not found among FED patients (i.e., AN, BN, BED, and FEDNOS), a post hoc test showed significant differences between BN and obese patients. This finding suggests that FEDs, although differing in symptomatology, are characterized by similar levels of psychological distress that are less pronounced in obese people, partially supporting the exclusion of obesity from FED psychopathological classification. Moreover, the potentially ego-syntonic nature of FED symptomatology occurring in patients with obesity, which may protect against experiencing high psychological distress, could explain the tendency to maintain excessive food consumption above their needs.

The second hypothesis, that the presence of a comorbid PD was associated with higher psychological distress in patients with FEDs, was also partially confirmed. As expected, co-occurring PDs were found in the majority of our sample of FED patients, with no significant differences in their occurrence among FED subtypes. Contrary to our expectations, our sample showed no differences in psychological symptoms between FED patients with or without comorbid PDs, with the exception of depressive symptoms. Therefore, this finding did not support the hypotheses according to which the presence of a comorbid PD would impact psychological distress and FED symptoms [57,58]. The results instead suggest a relationship between PDs and depression, which is consistent with evidence of a strong impact of PD on mood [59].

The third hypothesis, that personality pathological traits and purging symptoms predicted psychological distress in FED patients, was fully confirmed. Due to the high occurrence of PDs in our sample, mean scores obtained on the five personality domains (i.e., negative affectivity, detachment, antagonism, disinhibition, and psychoticism) were in the range of average scores found in other clinical samples [49]. Our findings suggested that personality dimensions as negative affectivity and detachment, together with purging symptoms, predicted psychological distress in FED patients. In FED patients, particularly those with BN, negative affectivity—linked to depressive symptoms—and detachment could be related to purging symptoms, creating a vicious circle of mutual reinforcement between dysfunctional personality traits and purging symptoms. These findings could explain why BN patients tend to experience greater distress and suggest that considering personality dimensions rather than categorical PD diagnoses should be considered, since they seem to have an impact on the psychological distress of FED patients.

Finally, the fourth hypothesis, that emotion regulation mediated the effect of self-esteem on purging symptoms in FED patients, was also confirmed. The results of mediation analysis showed that low self-esteem had no direct effect on purging symptoms, but when it is mediated by emotional dysregulation, the mediated effect becomes significant. Since emotional dysregulation was also an independent predictor of purging symptoms, findings suggest that it is essential to monitor patients’ vulnerability in managing emotional states in order to impact the course of the disease. These results are consistent with a substantial body of literature [60,61] that has extensively examined the role of emotional regulation in the etiology and maintenance of FEDs. It is widely recognized that deficits in emotion regulation capacity constitute a core socio-emotional vulnerability, significantly contributing to both the onset and persistence of disordered eating behaviors. Meta-analytic evidence [7,61] indicates that individuals with AN, BN, and BED frequently exhibit a dispositional tendency toward emotion dysregulation. These findings suggest that maladaptive emotion regulation strategies are closely linked to the severity of FED symptoms. Building on this, Peterson et al. [62] emphasized the pivotal role of emotion regulation in predicting both treatment outcomes and symptom severity, identifying this capacity as a crucial target within the therapeutic process for individuals with FEDs. Furthermore, these findings may be interpreted by considering that emotional dysregulation may operate not only at a conscious and explicit level, but also through implicit regulatory processes. In this perspective, implicit emotion regulation can be conceptualized as underlying automatic processes contributing to the modulation of emotional states and to the maintenance of maladaptive behaviors, such as purging, particularly in individuals characterized by high negative affectivity and interpersonal detachment [63,64].

Taken together, these findings suggest a complex interaction between certain personality dimensions and FED symptoms in determining the psychological distress of FED patients. Our results confirmed that psychological distress is higher in FED patients than in people with severe overweight, supporting the exclusion of obesity in the diagnostic classification of FEDs from the DSM-5-TR. Moreover, they suggest that the presence of a personality disorder per se does not significantly compromise mental health in FED patients. Rather, some personality dimensions, such as negative affectivity and detachment, together with purging symptoms, appear to predict psychological distress in individuals with FEDs. In particular, patients’ vulnerability in experiencing and regulating negative emotions mediates the impact of low self-esteem on purging symptoms, consequently increasing their overall psychological distress [65].

The present research has several limitations that should be considered when interpreting the results. First, the sample size was limited to female patients and was not equally distributed among FED subtypes, which limits the generalizability of the findings to the target population. Second, the assessment was conducted using only self-reported questionnaires, which considers only the patient’s perspective of their psychological functioning. Third, emotional dysregulation, low self-esteem, and purging symptoms were assessed using the EDI-3 component scales rather than with a measure assessing the general constructs, which may limit the interpretation of results. Moreover, since minors were assessed using the same survey used for adult participants, the potential impact of developmental heterogeneity and self-report bias in minors should be carefully considered. Finally, the lack of a valid and reliable instrument that integrates the assessment of both implicit and explicit components of emotional regulation did not allow an in-depth investigation of the role of emotion regulation in the onset and maintenance of FED symptoms. Future studies should consider recruiting a larger and equally stratified sample of individuals affected by FEDs, applying both self-report and observer-rated measures, and integrating the evaluation with a multi-method approach that includes the use of other-report questionnaires together with quantitative and qualitative textual analyses [66,67,68] and other process-sensitive analytic approaches [69,70,71]. Moreover, they should consider including the evaluation of implicit components of emotion regulation, which have been extensively shown to impact mental well-being and adaptation [72,73,74,75]. Finally, future research should acknowledge the need for longitudinal designs to test mediation pathways.

## 5. Conclusions

In light of the findings from this study, it is necessary to reflect on the importance of considering specific aspects of personality functioning when planning tailored treatment with FED patients. Particular attention should be paid to how emotions are experienced, understood, and managed by the patient [25,56,76,77]. However, limiting therapeutic intervention to the explicit component of emotional regulation could lead to the concrete risk of overlooking the impact that implicit components of emotion regulation can have on the symptomatology of FEDs. With particular reference to purging symptoms, understanding the role of implicit emotion regulation strategies, such as acting out, passive aggression, and splitting of self-image, could provide important insights into how patients’ defensive functioning influences the occurrence of FED symptoms and how to intervene for a more effective treatment [78,79].

## Figures and Tables

**Table 1:** Differences between FEDs in psychological maladjustment (*N* = 124).

	*N*	*df*	*χ* ^2^	*p*
Ineffectiveness	29	4	7.50	0.112
Interpersonal Problems	41	4	13.08	0.011 *
Affective Problems	23	4	16.29	0.003 **
Overcontrol	24	4	17.60	0.001 ***
Global Psychological Maladjustment	7	4	15.45	0.004 **

Note: Kruskal–Wallis test; * *p* < 0.05; ** *p* < 0.01; *** *p* < 0.001.

**Table 2:** Frequency of personality disorders among patients with Feeding and Eating Disorders (*N* = 124).

	Personality Disorder					
FED	Absent		Present		Total	
	N	%	N	%	N	%
AN	12	9.7	17	13.7	29	23.4
BN	18	14.5	23	18.6	41	33.1
BED	8	6.4	15	12.1	23	18.6
OSFED	2	1.6	5	4.0	7	5.6
Obesity	10	8.1	14	11.3	24	19.4
Total	50	40.3	74	59.7	124	100.0

**Table 3:** Means and standard deviations of personality domains in FED patients (*N* = 124).

Personality Domain	*X*	*SD*	Minimum	Maximum	Clinical Sample ^a^	Δ *(X* vs. Clinical Sample)
Negative affectivity	1.64	0.44	0.57	2.58	1.41	+0.23
Detachment	1.39	0.53	0.16	2.29	1.26	+0.13
Antagonism	0.66	0.40	0.15	1.82	0.71	−0.05
Disinhibition	1.27	0.46	0.48	2.49	1.31	−0.04
Psychoticism	1.04	0.63	0.00	2.34	0.94	+0.10

^a^ = Reference to other clinical samples (Miller et al., 2022) [49].

**Table 4:** Linear multiple regression for personality and purging symptoms predicting psychological distress (*N* = 124).

Personality Domain	*β*	*SE*	t	*p*
Intercept	8.47	15.62	0.54	0.594
Negative affectivity	75.01	13.80	5.43	<0.001 ***
Detachment	−21.88	8.81	−2.48	0.023 *
Antagonism	−36.32	17.55	−2.07	0.052
Disinhibition	−3.16	18.66	−0.17	0.867
Psychoticism	2.02	9.58	0.21	0.835
Purging	0.25	0.11	2.38	0.028 *

Note: * *p* < 0.05; *** *p* < 0.001.

**Table 5:** Mediation model of low self-esteem on purging symptoms mediated by emotional dysregulation (*N* = 124).

Effect Type	Pathway	Β	SE	95% C.I.		β	z	*p*
				Lower	Upper			
Indirect	Low S-E ⟹ Emot Dysreg ⟹ Purging	0.122	0.612	0.002	0.242	0.107	1.992	0.046 *
Component	Low S-E ⟹ Emot Dysreg	0.595	0.092	0.415	0.776	0.502	6.468	<0.001 ***
	Emot Dysreg ⟹ Purging	0.205	0.098	0.013	0.397	0.212	2.093	0.036 *
Direct	Low S-E ⟹ Purging	0.014	0.116	−0.214	0.241	0.012	0.118	0.906
Total	Low S-E ⟹ Purging	0.136	0.102	−0.065	0.336	0.118	1.323	0.186

Note: * *p* < 0.05; *** *p* < 0.001; Low S-E = low self-esteem; Emot Dysreg = emotional dysregulation; Betas are completely standardized effect sizes.

## Data Availability

The data presented in this study are available on request from the corresponding author. The data are not publicly available due to privacy and ethical restrictions.
